# Dabigatran versus warfarin under standard or pharmacogenetic-guided management for the prevention of stroke and systemic thromboembolism in patients with atrial fibrillation: a cost/utility analysis using an analytic decision model

**DOI:** 10.1186/1477-9560-11-14

**Published:** 2013-07-17

**Authors:** Léon Nshimyumukiza, Julie Duplantie, Mathieu Gagnon, Xavier Douville, Diane Fournier, Carmen Lindsay, Marc Parent, Alain Milot, Yves Giguère, Christian Gagné, François Rousseau, Daniel Reinharz

**Affiliations:** 1 Département de médecine sociale et préventive, Faculté de Médecine, Université Laval, 1050, avenue de la Médecine, Québec, QC G1V 0A6, Canada; 2 Département de génie électrique, Faculté des Sciences et de génie, Université Laval, Québec, Québec, Canada; 3 Centre de recherche du centre hospitalier universitaire de Québec (CRCHUQ), Faculté de Médecine, Université Laval, Québec, Québec, Canada; 4 Département de médecine, faculté de Médecine, Université Laval, Québec, Québec, Canada; 5 Département de biologie moléculaire, biochimie médicale et pathologie, Faculté de Médecine, Université Laval, Québec, Québec, Canada

**Keywords:** Atrial fibrillation, Simulation, Cost-utility, Dabigatran etexilate, Warfarin, Anticoagulation, CYP2C9, VKORC1

## Abstract

**Background:**

Atrial fibrillation (AF) is the most common form of heart arrhythmia and a leading cause of stroke and systemic embolism. Chronic anticoagulation is recommended for preventing those complications. Our study aimed to compare the cost/utility (CU) of three main anticoagulation options: 1) standard warfarin dosing (SD-W) 2) warfarin dosage under the guidance of CYP2C9 and VKORC1 genotyping (GT-W) and 3) dabigatran 150 mg twice a day.

**Methods:**

A Markov state transition model was built to simulate the expected C/U of dabigatran, SD-W and GT-W anticoagulation therapy for the prevention of stroke and systemic thromboembolism in patients with atrial fibrillation over a period of 5 years under the perspective of the public health care system. Model inputs were derived from extensive literature search and government’s data bases. Outcomes considered were the number of total major events (thromboembolic and hemorrhagic events), total costs in Canadian dollars (1CAD$ = 1$US), total quality-adjusted life years (QALYs), costs/QALYs and incremental costs/QALYs gained (ICUR).

**Results:**

Raw base case results show that SD-W has the lowest C/U ratio. However, the dabigatran option might be considered as an alternative, as its cost per additional QALY gained compared to SD-W is CAD $ 4 765, i.e. less than 50 000, the ICUR threshold generally accepted to adopt an intervention. At the same threshold, GT-W doesn’t appear to be an alternative to SD-W. Our results were robust to one-way and multi-way sensitivity analyses.

**Conclusion:**

SD-W has the lowest C/U ratio among the 3 options. However, dabigatran might be considered as an alternative. GT-W is not C/U and should not currently be recommended for the routine anticoagulotherapy management of AF patients.

## Introduction

Atrial fibrillation (AF) is the most common form of heart arrhythmia. Its prevalence exceeds 2% in the general population of individuals who are 40 years old and more and increases with age from 0.1% in 50 years old patients to 10%-15% in those who are more than 80 years old [1]. The number of AF patients is expected to increase as the result of population ageing [1]. AF is the leading cause of stroke and is also associated with a high risk of systemic embolism [2,3]. Thus, long-term anticoagulation therapy is recommended in all patients with AF. Vitamin K antagonists such as warfarin are currently the most prescribed oral anticoagulants with, in the USA only, around 31 million prescriptions filled each year [4].

By depleting active vitamin K, warfarin therapy reduces the risk of stroke by about 60 percent in patients with AF [2]. However, warfarin has a narrow therapeutic range and is not devoid of adverse reactions: hemorrhages or thromboses occur in 6 to 39% of patients annually and are mainly related to the effectiveness of anticoagulation [5-7].

Yet, there is a large inter-individual heterogeneity in the optimal warfarin dosage required to achieve a therapeutic effect (inter-patients variability varies by a factor of 20) [8-10]. This optimal dosage is defined as a 2 to 3 score in the international normalized ratio (INR) score of the prothrombin time, as a score below 2 increases the risk of thrombotic events and a score above 3 of hemorrhagic events. Maintaining patients in the therapeutic range is therefore of utmost importance. Close INR monitoring is needed to reduce the risk of bleeding and thromboembolism [8-10].

Inter-patient variability in plasma warfarin concentration is dependent on variables such as age, diet, drug interactions, and liver function but also on some genes that affect the metabolism of the drug [11]. The most important known genes in the pharmacokinetics of warfarin are CYP2C9 (the gene coding for cytochrome P450 2C9) and VKORC1 (a gene coding for the vitamin K epoxide reductase complex subunit). Genetic variation in these two genes account for approximately 30% to 50% of the variance in warfarin concentration between individuals [9,10,12]. Adjusting the first doses of warfarin under the guidance of CYP2C9 and VKORC1 alleles determination reduces the risk of bleeding and thromboembolic complications [13]. Indeed, the US Federal Drug Administration (FDA) suggested, in August 2007, to update the warfarin information sheet to include information on the possibility of CYP2C9 and VKORC1 pharmacogenetic testing [14].

Several new drugs have been developed recently that represent a new strategy in the fight against thromboembolism in FA patients. Dabigatran is the first of these new products and constitutes the main alternative to warfarin presently. Dabigatran exerts its anti-thrombotic effect by binding to thrombin, which prevents the conversion of fibrinogen to fibrin. It has been shown to be associated with a lower occurrence of systemic embolism and stroke than warfarin and does not require a close laboratory monitoring [15,16]. However, because of its higher price, some health authorities still consider dabigatran as an exception drug that requires an authorization prior to its prescription for patients covered by public health insurances.

Economic studies have shown that dabigatran is expected to be a C/U option under an acceptability threshold of 50 000$/QALY [17-22], while results on the comparison between warfarin standard dosing (SD-W) and warfarin genetic-guided dosing (GT-W) remain inconclusive [23-26]. Only one study has compared the 3 strategies (dabigatran 150 mg, SD-W and GT-W). It concluded that dabigatran 150 mg is a C/U option, under the perspective of the acceptability threshold of 50 000$/QALY. However, this study did not consider all major events related to anticoagulation and used a time horizon poorly supported by data [21]. Our study was conducted to evaluate the expected C/U of these anticoagulation options under the perspective of the public health care system in order to provide elements in support to the decision making process by health authorities.

## Methodology

### Modeling and event probabilities

**Figure 1:**
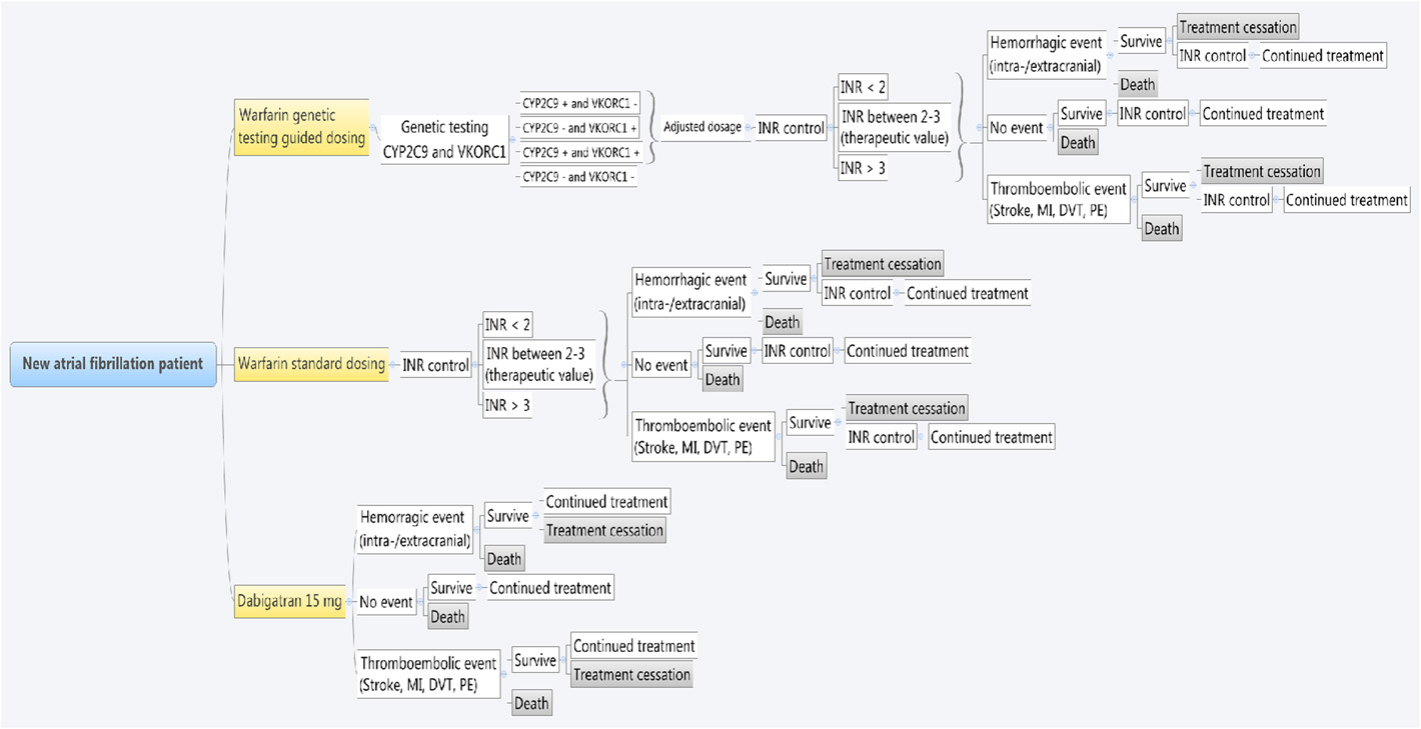
Markov state transition model.

A Markov state transition model was built to simulate the C/U of anticoagulation therapy for a virtual population of 10 000 individuals with a new diagnosis of AF under the perspective of the public health care system (Figure 1). The virtual population consisted of new AF patients with a mean age of 64 years (sd. = 8), who never had a previous stroke and who didn’t have a contraindication to anticoagulation therapy.

Outcomes considered were major hemorrhagic and thromboembolic events per 100 person-years, direct medical costs and quality-adjusted life-years (QALY). The model consists of daily cycles starting at the first day of anticoagulation treatment and ending 5 years later or at the death of an individual. The 5 years time horizon was chosen in order to take into account the chronicity of anticoagulation therapy in AF while avoiding to speculate about the long term effects of dabigatran.

Input parameters were retrieved from an extensive literature search of guidelines on anticoagulation in FA patients, and peer-reviewed published studies prioritized according to the following order: Quebec, other provinces of Canada, United States of America (USA), Europe and Australia.

The model begins by presenting the following three options: 1) SD-W; 2) GT-W; and 3) dabigatran 150 mg twice per day. Dabigatran was chosen among the new oral anticoagulants because it is the first one that has been approved in Canada and the first one that has been included in drug lists of the Canadian provincial public insurance schemes.

In the warfarin options (SD-W and GT-W), treatment monitoring is performed by measuring the international normalized ratio (INR). INR may be below (<2), within (between 2 and 3), or above (> 3) the therapeutic range. In the SD-W base case scenario, the time spent in each category (below, within and above) was based on the results of the RE-LY clinical trial comparing dabigatran and warfarin [16], while in the GT-W option, it was calculated using data from Anderson et al. 2007 [13]. We assumed that after one year 100% of individuals, whatever the warfarin group they belong to, had reached a stable maintenance dose [27]. The proportion of patient time spent in each INR category (below, within and above) was considered to be similar for both groups [27]. In the dabigatran option, it was assumed that no laboratory monitoring was required.

**Figure 2:**
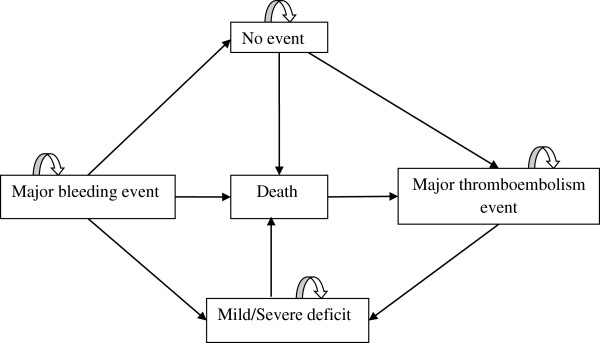
Diagram of Markov health states transition.

According to event probabilities [16,17,28,29], patients move into the Markov model through the following health states: no major event, major hemorrhagic event, major thromboembolic event, and death (Figure 2).

Major thromboembolic and hemorrhagic events were considered according to the definition of Fihn et al. [30]. Hemorrhages were classified into two categories: 1) intracranial (intracerebral and subdural hematoma) and 2) extracranial. We assumed that all extracranial hemorrhages were gastrointestinal as they constitute the majority of extracranial hemorrhages related to anticoagulant therapy regardless of the type of treatment [28,31]. Major thromboembolic events considered were strokes, myocardial infarctions (MI), deep vein thromboses (DVT) and pulmonary embolisms (PE). Following the occurrence of a major event, the model assumed that an individual visits the emergency room and that he has a defined probability of being hospitalized for a short or a long period of time. In addition, the model considers the probability of survival and of death after each event as well as the probability to have mild or severe post-event sequelae (see Table 1). It also considers a complete cessation of anticoagulant therapy in case of intracranial hemorrhage and a one month cessation in case of gastrointestinal bleeding [26].

**Table 1:** Model input parameters

**Parameter**				**Baseline**	**Range for sensitivity analysis**	**Distribution**	**Reference(s)**
**Event probabilities**							
Percentage of INR time in therapeutic range (2-3) in warfarin usual standard dosing				64%	55-69%	Beta	[16]
Proportion of INR time range below therapeutic range (<2)				54%	45-60%		[13]
Percentage of increasing of TTR by warfarin pharmacogenetic guided dosing				7.3%	0-30%		[13]
Risk of major bleedings INR in therapeutic range				1.4%	0.9-2.3%		[32,33]
Relative risk of major bleedings INR above therapeutic range				1	-	Fixed (reference)	
Relative risk of major bleedings INR below therapeutic range				4.7	3.57-10	Log-normal	
Relative risk of major hemorrhagic event dabigatran 150 mg vs warfarin				0.93	0.81-1.07		[16,17,28,29]
Major hemorrhagic events (warfarin treatment)		Proportion intracranial		42%	20-45%	Beta	[24,31]
		Proportion extracranial		58%	55-80%		[31]
Proportion major hemorrhagic events Dabigatran 150 mg		% Intracranial		12.6%	6.3-13.4%		[14,18-20]
		% Extracranial		90.4%	86.6-93.7		
Risk of major thromboembolic events INR in therapeutic range				2.4%	1.2-4.9		[32,34]
Relative risk of major thromboembolic events INR above therapeutic range				3.5	2.8-44	Log normal	
Relative risk of major thromboembolic events INR below therapeutic range				0.9	0.6-1.3		
Relative risk of major thromboembolic events dabigatran 150 vs warfarin		Stroke and systemic embolism		0.66	0.53-0.82		[16]
		MI		1.38	1-1.91		
		PE		1.61	0.76-3.42		
Major thromboembolic events (warfarin treatment)		% Stroke		52.5%		Fixed	[35]
		% Myocardial infarctus (MI)		12.5%			
		% Pulmonary embolism (PE)		30%			
		% Deep venous thrombosis (DVT)		5%			
Complications	Major hemorrhagic event	intracranial	No deficit	8%			[31,36]
			Mild deficit	16%			
			Severe deficit	34%			
			Death	42% (first month)			
		Extracranial		2%			[31]
	Major thromboembolismevent	Stroke		Month 1 : 8.3%			[24,36,37]
		Death		Months 2 and 3 : 5.6% per month			
		Severe deficit		40.2%			
		Mild deficit		42.5%			
		No deficit		9.1%			
		Death PE		12%			[38,39]
		Death DVT		6%			[38,40]
		Death IM		7%			[24]
Treatment discontinuation after a major event		Intracranial hemorrhage		100% during the entire period			[26] and expert opinion
		Extracranial hemorrhage		100% during 30 days			
**Costs (CAD$)**							
Drug costs		Dabigatran 150 mg		3.20/day	1-5	Gamma	[41]
		Warfarin 5 mg		0.074/day	0.03-0.1		
		LMWH		27.90/5 days	-	Fixed	
INR monitoring (first year)		SD-W		8.06/month	5-12	Gamma	[42]
		GT-W		5/month	2-8		Assumption
INR monitoring (subsequent years)				4.03/month	2-6		Assumption
Genetic tests (CYP2C9 and VKORC1)				615	100-1000		[43,44]
One-time event treatment costs		Ischemic stroke, no deficit		845	500-100		[43,44]
		Ischemic stroke, mild deficit		23772	15000-40000		[43-45]
		Ischemic stroke, severe deficit		42620	30000-60000		[43-46]
		Intracranial hemorrhage (ICH), non deficit		1067	25000-50000		[43-45]
		ICH mild deficit		21218	15000-25000		[43-45]
		ICH severe deficit		36451	25000-50000		[43-45]
		Subdural hematoma		31942	20000-45000		[43-45]
		Extra cranial hemorrhage		8146	5000-12000		[43,44]
		DVT		2576	1500-4000		[43,44]
		PE		8799	5000-9000		[43,45]
		MI		7177	5000-15000		[43,45]
Post-event cost		Severe disability stroke/ICH		6259/month	3000-10000		[44-46]
		Mild disability stroke/ICH		1855/month	1000-3000		
**Health utilities**							
Warfarin no event				0.95	0.95-0.98	Beta	[21,47,48]
Dabigatran no event				0.95	0.95-0.98		[21,47,48]
Major bleeding		ICH	No deficit	0.51	0.15-0.60		[21,47,48]
			Mild deficit	0.75	0.70-0.90		
			Severe deficit	0.95	0.90-0.95		
		Extracranial		0.80	0.75-0.85		
Stroke		No deficit		0.95	0.90-0.95		[21,47,48]
		Minor		0.75	0.70-0.90		
		Severe		0.39	0.15-0.50		
MI				0.84	0.80-0.90		[21,47,48]
PE				0.76	0.70-0.90		[21,47,48]
DVT				0.84	0.80-0.90		[21,47,48]

### Costs

Costs included in the study are those of the public health care system. Only direct costs were estimated (Table 2).

Health services consumed along the course of an anticoagulation therapy relate primarily to services associated with prescription, warfarine monitoring and management of thromboembolic or hemorrhagic complications (deep vein thromboses, pulmonary embolisms, strokes, myocardial infarctions, intracranial, sub-dural and extracranial hemorrhages) [42,49-55] as well as the cost of follow-up in case of sequelae from a stroke or an intracranial hemorrhage.

The quantification of services consumed was based on the literature. In all cases, we considered for baseline values, recommendations by the Canadian guidelines on the management of thrombosis and anticoagulation [42,49-55]. For items not found in the guidelines, we used relevant economic studies published in peer-reviewed journals. Unit prices for services consumed were calculated from the administrative data of the Quebec public health care system. The lowest prices from the list of drugs covered by Quebec public healthcare insurance (*Régie d’assurance maladie du Québec (RAMQ))* were used to estimate the cost of outpatient medication, to which were added 6% for wholesalers as well as the pharmacist fees paid by RAMQ. Diagnosis related-groups (DRG) data were used to calculate the average cost of hospitalization to which were added the physician fees paid by RAMQ. The ministry of health SIFO data bank was used to calculate activity center unit prices for ambulatory care. These prices were increased to reflect the contribution of support activity centers to clinical services, using the direct method [56]. The rate base used for these sources was the values of the 2010-2011 fiscal year.

### Utilities

Utility scores used for each health states are presented in Table 1. They were retrieved from published studies [21,47,48]. Utility scores were then used to weight the time spent in each health state to produce QALYs.

### Simulation process and analysis

Simulations were carried out on the SCHNAPS platform simulator [57,58] that runs on the CLUMEQ network super computers. In the “individual sampling model (ISM)” simulation that was performed, each virtual individual is generated and has his own path in the simulation process. Simulations were repeated 1000 times with different virtual populations of 10 000 individuals. However, each of the 1000 different virtual population generated was used for the three options compared. The principal outcome measured was the incremental cost-utility (ICUR) ratio calculated using the difference in average individual cost over the 1000 simulations divided by the difference in average QALYs. We considered the literature that proposes that an option can be accepted as cost-effective if its ICUR is CAD$ 50 000/QALY or less [59]. An option was considered dominated if it was more costly and less effective (less QALY) in comparison to its alternative (strict dominance) or if it’s incremental cost-effectiveness ratio was greater than that of the next, more effective, and more expensive alternative (extended dominance). All costs and QALY were discounted at a baseline annual rate of 3%.

### Sensitivity analyses

Univariate and multiway probabilistic sensitivity analyses were performed using the parameters that were foreseen as having a possible impact on the outcomes (Table 1). One way sensitivity analyses were performed to evaluate the eventual impact of each single parameter on the results. We tested the minimum and the maximum values (from the 95% confidence intervals) for each of these variables. For the GT-W option, we tested several patient percentage times in therapeutic range (TTR) values in order to find the value for which GT-W would be cost-effective compared to SD-W. Subsequently, multi-way probabilistic sensitivity analyses using Monte Carlo simulations were performed using a virtual population of 5000 individuals. We assumed that event probabilities and utility scores followed a beta distribution, that costs followed a gamma distribution while relative risks were assumed to have a log-normal distribution [60]. A C/U acceptability curve [61] was then produced from 1000 Monte Carlo iterations in order to better define the joint uncertainty of the parameters on C/U ratios.

### Validation

The Markov state decision model and the parameterization were validated by two experts (MA, PM) in genetics and anticoagulotherapy.

The simulation process was validated at each step to ensure that the data generated matched expected data. This consisted in verifying that the number of events (major bleedings and major thromboembolisms) corresponded to their predefined expected occurrence according to the literature [49-54].

### Ethical approval

This project was approved by the Research Ethics Committee of Laval University.

## Results

Base-case C/U results are presented in Table 2. SD-W has the lowest raw C/U ratio. However, compared to SD-W, dabigatran increases QALYs by 0.2529 and increases the cost/individual by CAD$ 1205 for an ICUR of 4765/QALY gained. It is therefore under the commonly proposed threshold of 50 000$/QALY. GT-W is not C/U. Compared to SD-W, it increases QALYs only by 0.0085, and increases the cost/individual ratio by CAD$ 460 for an ICER of 54 118/QALY gained. It is also dominated by dabigatran (extended dominance).

**Table 2:** Base case results

**Option**	**Major bleeds/100 person-year**	**Major TE/100 person-year**	**Cost/patient (CAD $)**	**QALY/patient**	**Cost/QALY**	**∆ Cost** **CAD $**	**∆ QALY**	**ICUR**
Standard warfarin^*^ dosage	3.21	2.12	7 289	3.5368	2061			
Pharmacogenetic oriented warfarin dosage	3.09	2.126	7 749	3.5453	2186	460	0.0085	Dominated
Dabigatran 150 mg BID	2.872	1.813	8 494	3.7897	2241	745	0.2444	4 765

*Less costly option.

Sensitivity analyses show that the results were robust either in one-way or in probabilistic multiway sensitivity analyses. In one-way sensitivity analyses, dabigatran remains an alternative to the SD-W option if the threshold of 50 000$/QALY is socially considered as acceptable [59]. Compared to SD-W, GT-W is not C/U except when this strategy allows patients to have an average of 76.8% of patient TTR, i.e. 20% more than in the SD-W option in the first year (Table 3). This value was found as the threshold for GT-W to be cost-effective and the ICUR is then 3250/QALY gained which is less than the acceptability threshold of 50 000 CAD $ [59].

**Table 3:** One-way Sensitivity analysis results

**Parameter**	**Option**	**Cost/patient**	**∆ cost**	**QALY**	**∆ QALY**	**ICUR**
76.8% of Patient TTR with warfarin pharmacogenetic guided dosing	Warfarin standard	7289		3,5358		
	Warfarin genetic testing	7611	322	3,6348	0,099	3253
	Dabigatran 150	8494	883	3,7897	0,1549	5700
RR major hemorragic event dabigatran 150 vs warfarin = 0,81	warfarin standard	7289		3,5358		
	warfarin genetic testing	7749	460	3,5453	0,0085	Dominated
	Dabigatran 150	8142	393	3,8667	0,3214	2586
RR major hemorragic event dabigatran 150 vs warfarin = 1,07	warfarin standard	7289		3,5358		
	warfarin genetic testing	7749	460	3,5453	0,0085	Dominated
	Dabigatran 150	8879	1130	3,695	0,1497	9987
RR stroke dabigatran 150 VS warfarin = 0,82	warfarin standard	7289		3,5358		
	warfarin genetic testing	7749	460	3,5453	0,0085	Dominated
	Dabigatran 150	8765	1016	3,708	0,1627	8570
RR stroke dabigatran 150 VS warfarin = 0,53	warfarin standard	7289		3,5358		
	warfarin genetic testing	7749	460	3,5453	0,0085	Dominated
	Dabigatran 150	8298	549	3,8113	0,2755	3660
Cost genetic tests = CAD$ 100	warfarin standard	7289		3,5368		
	warfarin genetic testing	7330	41	3.5453	0,0085	Dominated
	dabigatran 150	8494	1164	3.7897	0,2444	4765
Cost genetic tests = CAD$ 1000	warfarin standard	7289		3,5368		
	warfarin genetic testing	8050	41	3.5453	0,0085	Dominated
	dabigatran 150	8494	1164	3.7897	0,2444	4765
Day cost of dabigatran 150 = 1CAD$	Dabigatran 150	5217		3.7897		
	Warfarin standard	7289	2072	3.5368	−0,2444	Dominated
	Warfarin genetic testing	7749	460	3.5453	−0,0085	Dominated
Day cost of dabigatran 150 = 5$	Warfarin standard	7289		3,5368		
	Warfarin genetic testing	7749	460	3.5453	0,0085	Dominated
	Dabigatran 150 mg	11182	1164	3.7897	0,2444	15393
Utility score dabigatran without event =0,98	warfarin standard	7289		3,5358		
	warfarin genetic testing	7749	460	3,5453	0,0085	Dominated
	Dabigatran 150	8494	745	3,902	0,3567	3290

**Figure 3:**
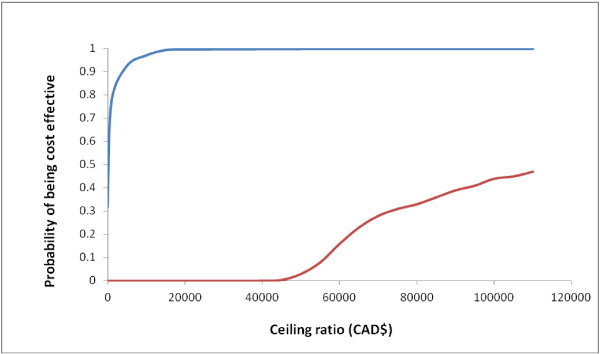
**Cost-utility acceptability curves.** Blue: The curve represents the probability of dabigatran to be cost-effective compared to warfarin standard dosing at various ceiling ratios thresholds. Red: The curve represents the probability of warfarin pharmacogenetic guided dosing to be cost-effective compared to warfarin standard dosing at various ceiling ratios thresholds.

In probabilistic multiway sensitivity analyses, dabigatran 150 mg remains the most C/U option. Compared to SD-W, it is dominant in 32% of iterations and cost-effective in 99.75% of iterations if the ceiling ratio threshold is fixed at CAD$ 50 000/QALY gained (Figure 3).

## Discussion

In this study, we evaluated through a simulation model the expected cost-utility of three anticoagulation options namely SD-W, GT-W and dabigatran 150 mg twice daily, over a 5 years’ time horizon and under a public health care perspective. Our results show that the dabigatran option is the most C/U option if the public health care system accepts to invest CAD $4 800 per additional QALY gained. This amount is lower than the 50 000 per QALY gained commonly proposed as the threshold to adopt an innovation in North-America [59]. Although our study has used a more complete model than several other C/E analyses, its results are consistent with the literature [17-20,22]. Nevertheless, we don’t know if the results could be generalized to all other NOACs as there is still a lack of data concerning the direct comparison between the different products on the market. Currently, only indirect comparisons exist on the efficacy and safety of the NOACs that show that they are effective compared to warfarin. Yet, when they are compared to each other, there is no difference in efficacy, although some differences in safety might exist [62,63].

The GT-W option was not cost/effective (if not dominated) compared to the two other options. This is in line with other studies that have compared warfarin treatments with or without genetic testing [23,25,26]. However, GT-W could be the most C/U option compared to SD-W and dabigatran150 mg, if the average patient time in the therapeutic range moves from 66.6% to 76.8% i.e. if it is 20% higher than in the SD-W option in the first year. However, this is a very ambitious objective to reach even in clinical trial conditions [13]. This result is close to that of You et al. [21] who showed that the GT-W option could become the most cost-effective option if the patient time in the therapeutic range was > 77%.

This study has some limitations. First, the key input parameters (events probabilities, INR control) comparing dabigatran vs. SD-W or SD-W vs. GT-W were taken from one single randomized controlled clinical trial. This issue could have decreased the ICUR since the effectiveness of dabigatran or GT-W may be overestimated by controlled clinical trials compared to the situation in real life. Nevertheless, we have done extensive sensitivity analyses in order to handle this problem.

The second limitation is the complexity of mapping the reality. Some simplifications and assumptions were inevitable in the modeling approach. For example, we did not consider minor events (bleedings and thromboembolism) that occur with anticoagulation therapy. This issue could have increased the ICURs.

Thirdly, our model did not consider patients’ adherence with medication in order to make a fairly comparison of the dabigatran option with the two others. Indeed, while longtime adherence for warfarin is available, there is still a lack of data on longtime adherence for dabigatran [64]. Taking into account the medication adherence would have led us to make assumptions about the adherence for dabigatran.

Finally, our model is limited by the consideration of only direct costs and one single perspective, i.e. the public healthcare perspective. The addition of the patients’ perspective could increase the ICUR especially in the case of SD-W that might require time and travel expenses for INR control.

Despite these limitations, the results of this study suggest that dabigatran 150 mg twice per day is a C/U alternative to SD-W. Its additional cost per QALY gained is considered as socially acceptable. GT-W is not C/U and should not now be recommended in routine management of warfarin anticoagulotheray in FA patients. However, our results produced in the Quebec/Canadian context (a quasi-exclusive public health care system) remain to be confirmed for other health care jurisdictions especially where the public system is not dominant.

## Competing interests

The authors declare that they have no competing interests.

## Authors’ contributions

All authors: Conception, design, acquisition and validation of data. MG, XD, DF: computer simulations. NL, DR, YG, FR, CG, AM, MP: analysis and interpretation of results. NL, DR, JD: Drafting the article. NL, DR, FR, AM, YG, MP: Critically revising of the article. All authors approved the final version of article.
